# Effect of a nutrition education intervention on mothers´ complementary feeding practices: a longitudinal study in two sub-county hospitals in Nyeri County, Kenya

**DOI:** 10.11604/pamj.2026.53.173.49709

**Published:** 2026-04-24

**Authors:** Lydiah Asiko Omondi, Joseph Kiplang´at Mutai, Anselimo Ouma Makokha

**Affiliations:** 1 Department of Environmental Health and Disease Control, School of Public Health, Faculty of Health Sciences, Jomo Kenyatta University of Agriculture and Technology, P.O. Box 62000-00200, Nairobi, Kenya,; 2 Centre for Public Health Research-KEMRI, P.O. Box 54840-00200, Nairobi, Kenya,; 3 Department of Food Science, School of Food and Nutrition Sciences, Jomo Kenyatta University of Agriculture and Technology, P.O. Box 62000-00200, Nairobi, Kenya

**Keywords:** Complementary feeding, nutrition education, dietary diversity, meal frequency, longitudinal study

## Abstract

**Introduction:**

sub-optimal complementary feeding remains a major contributor to child undernutrition during the first 1,000 days of life in low- and middle-income countries. Nutrition education is widely promoted to improve infant and young child feeding practices, but evidence from longitudinal facility-based interventions in Kenya is limited.

**Methods:**

a longitudinal intervention study was conducted among 451 mothers (intervention n = 220; control n = 231) of children aged 6-23 months attending two level-IV hospitals in Nyeri County, Kenya. The study sites were randomly selected using a simple randomization procedure involving an unbiased coin toss, ensuring each of the two eligible sites had an equal probability of selection. The “head” and the “tail” were assigned to the intervention and control sites, respectively. Participants meeting the inclusion criteria were then recruited from the site that was randomly assigned during their specified clinic visits. Mothers attending Karatina Sub-County Hospital received quarterly nutrition education sessions reinforced with cooking demonstrations over nine months, while those attending Othaya Sub-County Hospital received routine health education. Complementary feeding practices were assessed using World Health Organization/United Nations Children´s Fund infant and young child feeding indicators. Data were collected at baseline and at 3, 6, and 9 months. Data were collected using structured questionnaires and analyzed using the Statistical Package for Social Sciences (SPSS) v24. Proportions, percentage change, net effect of intervention, odds ratios (OR) with 95% confidence interval (CI), and Chi-square (χ^2^) tests were used; significance was set at P ≤ 0.05.

**Results:**

at three months, the intervention group showed significantly greater improvements in egg and flesh food consumption, breastfeeding, minimum meal frequency, and minimum dietary diversity compared with the control group. The net effect of intervention for egg and flesh food consumption was +51.6% (odds ratio 6.17; 95% confidence interval 4.02-9.46; P < 0.001). Improvements in egg and flesh food consumption, breastfeeding, and minimum meal frequency were sustained at six and nine months. Reduction in zero vegetable and fruit consumption was greater in the control group at later follow-up points.

**Conclusion:**

a structured nutrition education intervention reinforced with cooking demonstrations improved several key complementary feeding practices over nine months. Integrating such interventions within routine maternal and child health services may contribute to improved infant and young child feeding outcomes.

## Introduction

Undernutrition remains a major global public health challenge, especially in low- and middle-income countries [1,2]. It contributes to over half of under-5 mortality and is worsened by food insecurity, poverty, and illiteracy [3,4]. The “first 1,000 days” period from birth to 24 months is vital for optimal nutrition to support growth, development, and long-term health [5,6]. Poor feeding practices during this period can cause irreversible consequences [7].

A key intervention during this vulnerable period is complementary feeding (CF), defined as the process starting at six months of age when breast milk alone is no longer adequate to meet the nutritional requirements of infants, necessitating the introduction of solid, semi-solid, and liquid foods alongside continued breastfeeding up to 24 months or beyond. Optimal CF practices also include ensuring minimum dietary diversity (MDD) (provision of at least 4 out of 7 food groups), minimum meal frequency (MMF), minimum acceptable diet (MAD), and provision of other nutritious foods, including egg, flesh food, vegetable, and fruit consumption [8].

Despite established guidelines, sub-optimal complementary feeding practices remain common, especially in low- and middle-income countries (LMICs). Less than a quarter of children aged 6-23 months meet dietary diversity, meal frequency, or acceptable diet standards [9]. Studies in Southwestern Nigeria [10] found very low rates of children meeting MMF (33.6%), MDD (14.5%), and MAD (9.2%). In Seshego, South Africa, only 45.1% of caregivers introduced food at six months [11]. In Turkana County, Kenya, a region predominantly characterized by high poverty, cereal-based complementary foods are introduced either too early or too late in the first year of the infant´s life [12]. In Nairobi, Kenya, overall poor child feeding practices, including MDD, MMF, and MAD, were identified as a characteristic feature [13]. Although nutrition education is widely used to promote optimal infant and young child feeding practices [14], there is limited longitudinal evidence in Kenya evaluating its effectiveness on specific complementary feeding indicators such as dietary diversity, meal frequency, and consumption of nutrient-dense foods [15]. This study, therefore, assessed the effect of a targeted nutrition education intervention on mothers´ complementary feeding practices in Nyeri County.

Nyeri County, located in the central region of Kenya, has made progress in maternal and child health services; however, suboptimal infant and young child feeding practices remain a concern in the region. Although health facilities provide routine maternal and child health services [16], including growth monitoring and nutrition counselling, gaps persist in the consistent uptake and application of recommended complementary feeding practices at the household level. Socio-economic factors, caregiver knowledge, cultural feeding practices, and access to diverse foods may influence feeding behaviours among mothers with young children. Understanding how targeted nutrition education delivered through health facilities influences complementary feeding practices in this context is therefore important for informing local nutrition interventions and strengthening child nutrition programs within the county. This study aimed to assess the effect of nutrition education on mothers´ complementary feeding practices among children aged 6-23 months attending Karatina and Othaya Sub-County Hospitals in Nyeri County, Kenya.

## Methods

**Study design and setting:** this was a longitudinal intervention study conducted in Nyeri County, Kenya, between May 2021 and March 2022. The study was implemented in two public level-IV health facilities: Karatina Sub-County Hospital (intervention site) and Othaya Sub-County Hospital (control site). The two facilities are approximately 40 km apart, serve culturally comparable populations with similar dietary practices, and both have high uptake of maternal and child health services.

**Study population:** the study population comprised mothers or primary caregivers with children aged 6-23 months attending maternal and child health clinics at the selected facilities. Eligible participants were those who were residents of the study area, attended the selected facilities during the study period, and provided informed consent. Mothers or caregivers with children outside the specified age range, those who were seriously ill, or those unwilling to participate were excluded from the study. The study sites were assigned to either the intervention or control group using simple randomization through an unbiased coin toss, ensuring equal probability of allocation. The sample size was calculated using Cochran´s formula [17] for comparing two proportions.


$$n=\frac{{\left(Z_{1-\alpha /2}\sqrt{2P\left(1-P\right)}+Z_{1-\beta }\sqrt{P_{1}\left(1-P_{1}\right)+P_{2}\left(1-P_{2}\right)}\right)}^{2}}{{\left(P_{1}-P_{2}\right)}^{2}}$$

Therefore, n= ("1.96 x"√0.1638+1.282" x “√0.1588^2^)/”(0.96-0.86)^2^”. Adjusted 10% for loss to follow-up. Where: Z_1-α/2_= standard errors from the mean corresponding to a 95% confidence interval (1.96); Z_1-β_= power of the test (1.282); P1 = proportion in intervention group; P2 = proportion in comparison group; P=P1+P2/2.

Statistical significance was assessed at a 95% confidence level, with P-values < 0.05 considered statistically significant. A minimum of 171 participants per study arm was obtained, and after adjusting for a 10% potential loss to follow-up, the sample size was increased to 190 participants per arm. At baseline, 220 participants were enrolled in the intervention arm at Karatina Hospital and 231 in the control arm at Othaya Hospital. Participants were recruited consecutively from maternal and child health clinics until the required sample size per arm was achieved. Mothers in the intervention group received targeted nutrition education on appropriate complementary feeding practices, including dietary diversity, meal frequency, breastfeeding, and the inclusion of nutrient-dense foods. The education was delivered through routine maternal and child health clinics using standardized materials and education sessions. The control group received the standard routine care provided at the health facilities without additional targeted nutrition education.

**Data collection:** socio-demographic variables such as marital status, education, age, occupation, and religion were collected through a face-to-face interview. The independent variable was targeted nutrition education delivered for approximately 45 minutes, which emphasized the following: continued breastfeeding for at least 2 years, initiation of complementary food, increasing the number of feedings with age, increasing food density (consistency), food quality with age, increasing feedings when the child is ill, and diet diversification. The intervention comprised quarterly (every 3 months) group nutrition education sessions (30-45 minutes) grounded in social cognitive theory [18], plus 1-hour cooking demonstrations using locally available foods. Content emphasized timely CT at 6 months, dietary diversity, age-appropriate frequency, continued breastfeeding ≥ 2 years, and inclusion of eggs, flesh foods, fruits, and vegetables; demonstrations followed validated Ministry of Health (MoH)/Ministry of Agriculture (MoA) complementary-feed recipes [19]. Control facilities continued with routine health education sessions only. Demographic data and complementary feeding data were stored in a password-protected computer. Before the commencement of data collection, the questionnaire was pre-tested to ensure validity and reliability. This was conducted on thirty participants at Mukuruweini Sub-County hospital, a different level-4 health facility within Nyeri County. Trained nutrition research assistants collected the data.

**Definitions:** the dependent variables were EFF (defined as the proportion of children who consumed EFF the previous day over total number of children); ZVF (proportion of children who did not consume vegetables and fruits the previous day over the total number of children); MMF (proportion of children who consumed solid, semi-solid or soft foods minimum 2 meals per day (for an infant aged 6-8 months) plus minimum 3 meals per day (for an infant/child aged 9-23 months) during the previous day over the total number of children; MDD (proportion of children who consumed foods and beverages from at ≥ 4/7 defined food groups during the previous day over the total number of children); and MAD (composite of MMF and MDD) [20]. Visual aids (flip charts, food models) supported recall during intervention sessions.

**Statistical analysis:** data were entered and analysed using SPSS version 24. Socio-demographic characteristics were summarized using frequencies and percentages. Statistically significant differences were determined for categorical variables using chi-square tests for both the intervention and control groups. Changes in complementary feeding indicators from baseline to each follow-up point (3, 6, and 9 months) were calculated within each group, and the net effect of intervention was determined as the difference in percentage change between groups. Odds ratios with 95% confidence intervals were computed to compare outcomes between groups. Statistical significance was assessed at a 95% confidence level, with P-values < 0.05 considered statistically significant.

**Ethical consideration:** the study obtained ethical approval from the Ethical Review Committee of the Kenyatta National Hospital/University of Nairobi (approval number P585/10/2020, dated 12^th^ March 2021). A research permit was obtained from the National Commission for Science, Technology and Innovation (approval number 770282, dated 17^th^ March, 2021) and from Nyeri County Health Authorities (reference CGN/HEALTH/HRM/5/VOL.II, dated 7^th^ April, 2021). Written informed consent was obtained from all participants before data collection. Participant confidentiality was maintained by unique study identification and restricted data access.

## Results

**Participant characteristics:** a total of 451 mother-child pairs were enrolled at baseline, with 220 participants in the intervention arm (Karatina Sub-County Hospital) and 231 in the control arm (Othaya Sub-County Hospital). Baseline socio-demographic characteristics were generally comparable between the two groups, including maternal age, education level, occupation, and religion. A significantly higher proportion of mothers in the intervention arm were married compared with the control arm (84.1% vs 66.7%, P < 0.001) (Table 1).

**Table 1:** socio-demographic characteristics of mothers, Nyeri County, 2022

Variables		Intervention (n=220)	Control; (n=231)		
		n (%)	n (%)	χ^2^	P-value
**Marital status**	Married	185 (84.1%)	154 (66.7%)	26.861	**0.001**
	Single	32 (14.5%)	65 (28.1%)		
	Divorced	1 (0.5%)	2 (0.9%)		
	Separated	1 (0.5%)	9 (3.9%)		
	Other	1 (0.5%)	1 (0.4%)		
**Highest level of education**	No formal education	0 (0%)	1 (0.4%)	2.994	0.560
	Primary	36 (16.4%)	31 (13.4%)		
	Secondary	124 (56.4%)	123 (53.2%)		
	Vocational	42 (19.1%)	53 (22.9%)		
	University	18 (8.2%)	23 (10%)		
**Age category**	Less than 20	7 (3.2%)	7 (3%)	0.317	0.850
	20-34	171 (77.7%)	175 (75.8%)		
	35-49	42 (19.1%)	49 (21.2%)		
**Occupation**	Employed	29 (13.2%)	35 (15.2%)	0.371	0.950
	Casual	28 (12.7%)	28 (12.1%)		
	Self-employment	86 (39.1%)	89 (38.5%)		
	Housewife	77 (35%)	79 (34.2%)		
**Religion**	Christian	218 (99.1%)	230 (99.6%)	3.055	0.220
	None	2 (0.9%)	1 (0.4%)		

A p -value ≤0.05 indicates a statistically significant difference between groups at baseline.

**Complementary feeding outcomes at 3 months:** at three months of follow-up, significant improvements were observed in several complementary feeding indicators in the intervention group compared with the control group (Table 2). The proportion of children consuming egg and flesh foods increased markedly in the intervention arm, with a net effect of intervention of +51.6% (odds ratio 6.17; 95% confidence interval 4.02-9.46; P < 0.001). Breastfeeding, minimum meal frequency, and minimum dietary diversity also showed significantly greater improvements in the intervention group. No significant between-group difference was observed for the minimum acceptable diet at this time point.

**Table 2:** effect of intervention on complementary feeding practices at the 3^rd^ month, Nyeri County, 2022

		Intervention			Control				
Key indicators	Baseline (%)	3 months (%)	% change	Baseline (%)	3 months (%)	% change	^g^NEI	Odds ratio (95% CI)	P-value
^a^EFF	19.1	76.5	57.4	9.52	15.3	5.78	**51.6**	6.17	0.001
^b^ZVF	43.2	46.5	3.32	43.6	44.5	0.9	**2.42**	0.28	0.001
^c^BF	93.2	96.0	2.82	91.8	90.3	-1.50	**4.32**	1.7	0.05
^d^MMF	67.0	92.3	25.3	67.0	69.5	2.5	**22.8**	0.07	0.001
^e^MDD	17.3	66.0	48.7	20.8	30.0	9.2	**39.5**	1.96	0.001
^f^MAD	15.9	43.0	27.1	20.6	31.0	10.4	16.7	1.17	0.308

a Egg flesh food, ^b^zero vegetable and fruit, ^c^breastfeeding, ^d^minimum meal frequency, ^e^minimum dietary diversity, ^f^minimum acceptable diet, ^g^NEI - net effect of intervention

**Complementary feeding outcomes at 6 months:** at six months, sustained improvements were observed in the intervention group for egg and flesh food consumption and breastfeeding compared with the control group (Table 3). The net effect of the intervention for egg and flesh food consumption was +30.0% (P < 0.001), while breastfeeding rates declined in the control group but remained stable in the intervention group (net effect +7.0%, P < 0.001). The reduction in zero vegetable and fruit consumption was greater in the control group than in the intervention group. Differences between groups for minimum dietary diversity, minimum meal frequency, and minimum acceptable diet were small and not statistically significant.

**Table 3:** effect of intervention on complementary feeding practices at the 6^th^ month, Nyeri County, 2022

		Intervention			Control				
Key indicators	Baseline (%)	6 months (%)	% change	Baseline (%)	6 months (%)	% change	^g^NEI	Odds ratio (95% CI)	P-value
^a^EFF	19.1	60	40.9	9.52	20.4	10.9	30.0	3.54	0.001
^b^ZVF	43.2	43.8	0.6	43.6	46.2	2.6	-2.0	0.26	0.001
^c^BF	93.2	93.4	0.2	91.8	85.0	-6.8	7.0	0.45	0.001
^d^MMF	67.0	94.1	27.1	67.0	64.2	-2.8	20.9	0.61	0.108
^e^MDD	17.3	43.2	25.9	20.8	47.1	26.3	-0.4	0.83	0.206
^f^MAD	15.9	42.2	26.3	20.6	43.7	23.1	3.2	0.84	0.244

a Egg flesh food, ^b^zero vegetable and fruit, ^c^breastfeeding, ^d^minimum meal frequency, ^e^minimum dietary diversity, ^f^minimum acceptable diet, ^g^NEI - net effect of intervention

**Complementary feeding outcomes at 9 months:** at nine months of follow-up, the intervention group continued to demonstrate significantly greater improvements in egg and flesh food consumption, breastfeeding, and minimum meal frequency compared with the control group (Table 4). The net effect of intervention was +31.1% for egg and flesh food consumption (P < 0.001), +13.9% for breastfeeding (P < 0.001), and +28.5% for minimum meal frequency (P = 0.021). Improvements in minimum dietary diversity and minimum acceptable diet were observed in both groups, with no statistically significant differences between arms. Reduction in zero vegetable and fruit consumption remained greater in the control group.

**Table 4:** effect of intervention on complementary feeding practices at the 9^th^ month, Nyeri County, 2022

		Intervention			Control				
Key indicators	Baseline (%)	9 months (%)	% change	Baseline (%)	9 months (%)	% change	^g^NEI	Odds ratio (95% CI)	P-value
^a^EFF	19.1	67.1	48	9.52	26.4	16.9	31.1	3.18	0.001
^b^ZVF	43.2	46.7	3.52	43.6	45.6	2.0	1.52	0.28	0.001
^c^BF	93.2	93.6	0.4	91.8	78.2	-13.5	13.9	0.61	0.001
^d^MMF	67.0	96.4	29.4	67	67.09	0.9	28.5	0.42	0.0207
eMDD	17.3	52.7	35.4	20.8	57.0	36.2	-0.8	0.81	0.161
^f^MAD	15.9	50.9	35.0	20.6	55.4	34.8	0.2	0.78	0.0957

a Egg flesh food, ^b^zero vegetable and fruit, ^c^breastfeeding, ^d^minimum meal frequency, ^e^minimum dietary diversity, ^f^minimum acceptable diet, ^g^NEI - net effect of intervention

## Discussion

This study aimed to determine the effect of nutrition education on mothers´ complementary feeding practices in Nyeri County, Kenya. The findings demonstrated that nutrition education significantly improved key complementary feeding indicators in the intervention group, including dietary diversity, meal frequency, and consumption of nutrient-dense foods such as eggs and flesh foods. Improvements were also observed in breastfeeding practices, although reductions in zero vegetable and fruit consumption were more favourable in the control group. The socio-demographic characteristics observed in this study, including the predominance of married mothers and participants aged 20-34 years, are consistent with findings from other studies in sub-Saharan Africa, where women in this age group are typically the primary caregivers responsible for infant feeding [21-23]. The level of maternal education observed also aligns with previous studies, suggesting that secondary education may play a role in enhancing caregivers´ ability to adopt recommended feeding practices [24-26].

The significant improvement in dietary diversity following the intervention is consistent with evidence from similar studies conducted in Ethiopia and Uganda, where nutrition education resulted in marked increases in the proportion of children meeting minimum dietary diversity thresholds [6,27,28]. These findings are supported by systematic reviews demonstrating that nutrition education interventions can effectively improve infant and young child feeding practices [14,29-31]. However, some studies have reported no significant improvements in dietary diversity [32-34], indicating that contextual factors such as food availability, cultural practices, and socio-economic status may influence the effectiveness of such interventions. Similarly, the observed improvement in meal frequency in this study is consistent with findings from Uganda, where significant increases in the proportion of children meeting minimum meal frequency were reported following nutrition education interventions [35].

The increased consumption of animal-source foods, particularly eggs and flesh foods, in the intervention group further highlights the effectiveness of nutrition education in promoting nutrient-dense diets among young children. Comparable findings have been reported in studies conducted in Ethiopia and Uganda, where consumption of animal-source foods improved following targeted nutrition education interventions [35,36-38]. These foods are critical sources of essential micronutrients necessary for optimal child growth and development. In addition, the observed improvements in breastfeeding practices reinforce the importance of integrating breastfeeding promotion within complementary feeding interventions [22,29,38].

The findings of this study have important public health implications. They demonstrate that structured nutrition education delivered through routine maternal and child health services can significantly improve complementary feeding practices. This suggests that strengthening facility-based nutrition education programs could be an effective strategy for addressing sub-optimal infant and young child feeding practices in similar settings. However, the limited improvement in vegetable and fruit consumption indicates the need for complementary interventions that address food availability, accessibility, and affordability at the household level.

**Limitations:** the assessment of feeding practices relied on maternal recall, which may be subject to recall and social desirability bias. Nevertheless, efforts were made to minimize this through probing during interviews and by collecting baseline data before the intervention. A key strength of the study was its longitudinal design with a nine-month follow-up period, which allowed for the assessment of changes over time and strengthened the inference of a causal relationship between the intervention and observed outcomes.

## Conclusion

This study demonstrated that a structured nutrition education intervention was associated with improvements in key complementary feeding practices over 9 months, particularly in dietary diversity, meal frequency, and the consumption of nutrient-dense foods such as eggs and flesh foods. These findings suggest that facility-based nutrition education can play an important role in improving infant and young child feeding practices in similar settings.

### What is known about this topic

- Sub-optimal complementary feeding practices contribute substantially to child undernutrition in low- and middle-income countries;
- Nutrition education is commonly used to promote appropriate infant and young child feeding practices;
- Evidence on the sustained effects of facility-based nutrition education interventions in Kenya remains limited.

### What this study adds

- Facility-based nutrition education reinforced with cooking demonstrations improves key complementary feeding indicators;
- Sustained improvements were observed in egg and flesh food consumption, breastfeeding, and minimum meal frequency over nine months;
- Longitudinal follow-up provides evidence of medium-term benefits of nutrition education interventions.
